# Carotid Stiffness Assessment With Ultrafast Ultrasound Imaging in Case of Bicuspid Aortic Valve

**DOI:** 10.3389/fphys.2019.01330

**Published:** 2019-10-23

**Authors:** Guillaume Goudot, Tristan Mirault, Lina Khider, Olivier Pedreira, Charles Cheng, Jonathan Porée, Maxime Gruest, Xavier Jeunemaître, Mathieu Pernot, Emmanuel Messas

**Affiliations:** ^1^Physics for Medicine Paris, INSERM U1273, ESPCI Paris, CNRS FRE 2031, PSL Research University, Paris, France; ^2^VASC European Research Network, Centre de Référence des Maladies Vasculaires Rares, Hôpital Européen Georges-Pompidou, AP-HP, Paris, France; ^3^INSERM U970 PARCC, Paris Descartes University – USPC Sorbonne Paris Cité University, Paris, France

**Keywords:** bicuspid aortic valve, arterial stiffness, ultrafast ultrasound imaging, carotid, wall shear stress

## Abstract

**Aims:**

To compare the carotid stiffness and flow parameters by ultrafast ultrasound imaging (UF), in bicuspid aortic valve (BAV) patients to first-degree relatives (controls).

**Methods:**

BAV patients (*n* = 92) and controls (*n* = 48) were consecutively included at a reference center for BAV. Aortic valve and ascending aorta were evaluated by echocardiography. Common carotid arteries were evaluated by UF with a linear probe. A high frame rate (2,000 frames/s) was used to measure the pulse wave velocity (PWV). The arterial diameter change over the cardiac cycle was obtained by UF-Doppler imaging. This allowed us to measure the distensibility and the maximal rate of systolic distension (MRSD). The wall shear stress (WSS) was measured based on the same acquisitions, by analyzing blood flow velocities close to the carotid walls.

**Results:**

BAV patients had significantly larger aortic diameters (*p* < 0.001) at the Valsalva sinus and at the tubular ascending aorta but no larger carotid diameters. No significant differences were found in carotid stiffness parameters (distensibility, MRSD, and PWV), even though these patients had a higher aortic stiffness. Carotid stiffness correlated linearly with age and similar slopes were obtained for BAV patients and controls. No difference in carotid WSS was found between BAV patients and controls.

**Conclusion:**

Our results clearly show that the carotid stiffness and flow parameters are not altered in case of BAV compared with controls.

## Introduction

Bicuspid aortic valve (BAV) is associated with alterations of the aortic wall that lead to a higher risk of aortic aneurysm and acute aortic events (Verma and Siu, 2014). The decision to replace the ascending aorta by prophylactic surgery is currently only based on the aortic diameter (Aboyans et al., 2017). Due to early histological changes in the aortic wall, particularly affecting elastin fibers, it has been suggested that aortic stiffness may be a prognostic marker of dilatation (Nistri et al., 2008; Aquaro et al., 2017; Goudot et al., 2019a). The involvement of common carotid arteries in the aortic remodeling process in case of BAV remains controversial. Carotid dissections seem indeed to be part of the BAV spectrum (Schievink and Mokri, 1995) and Li et al. (2016) found a reduced arterial distensibility at the common carotid artery site. This finding was not confirmed by Santarpia et al. (2012) nor, more recently, by Kim et al. (2017) using tissue Doppler velocities. Nevertheless, common carotid arteries share a common embryological origin with the ascending aorta from the neural crest (Le Lièvre and Le Douarin, 1975; Majesky, 2007). More accessible to ultrasound imaging, their evaluation could therefore constitute a useful prognostic marker to predict acute aortic syndromes. More explorations are, however, required to ascertain the presence of a “carotidopathy” associated with the BAV.

Ultrasound imaging is the ideal tool for the follow-up of patients with BAV, due to its availability, its low cost, and its radiation free technology. Ultrafast ultrasound imaging (UF), using plane wave transmits with multiple inclinations and a sampling rate over 1,000 frames/s, allows to display the fine tissue displacements at high temporal resolution (Tanter and Fink, 2014) and Ultrafast Doppler, obtained by post-processing the same data, estimates precisely the tissue velocities (Bercoff et al., 2011). This new imaging modality gives access to local parameters of arterial stiffness, such as the pulse wave velocity (PWV) and the arterial wall distensibility. The PWV measurement with UF was initially presented and validated *in vivo* in healthy volunteers by Couade et al. (2011). A good agreement with other techniques was then established: distensibility using a high-resolution echo-tracking device (MyLab 70, ART. LAB, Esaote, Italy) (Marais et al., 2019) and the carotid-femoral PWV obtained by SphygmoCor^®^ (Mirault et al., 2015).

New biomarkers developed with UF, such as arterial stiffening over the cardiac cycle, may also help better define the arterial phenotype, as it has been done in the case of vascular Ehlers-Danlos syndrome (Mirault et al., 2015; Goudot et al., 2018).

In this work, we aimed at evaluating the changes in carotid biomechanical properties associated with BAV. We also evaluated the influence of the wall shear stress. As systematic screening of BAV in the family of the patient is performed in our center, we collected data from non-BAV relatives using the same protocol. This method allowed us to compare cases with controls sharing the same genetic background but without BAV.

## Materials and Methods

### Population

This study is a cross-sectional study of 140 consecutive patients undergoing dedicated consultation, between December 2017 and December 2018 at the European hospital Georges-Pompidou, a reference center for BAV disease. Patients with BAV have been prospectively evaluated at the National Reference Centre for Rare Vascular Diseases in a dedicated consultation. First-degree healthy relatives, i.e., with a tricuspid aortic valve, were used as controls. The ethical committee approved this study and patients signed a written informed consent form (CPP Île-de-France VI, n°2017-A01508-45). Confirmation of BAV was retained in case of the short-axis view of the aortic valve with the presence of only two functional cusps. First-degree relatives were also prospectively included and were considered as controls if a tricuspid aortic valve was found. In case of doubt about the diagnosis, the cardiac ultrasound loops were proofread by an expert physician, and a cardiac magnetic resonance imaging (MRI) was requested to evaluate the morphology of the aortic valve more precisely.

### Transthoracic Cardiac Ultrasound

Transthoracic echocardiography was performed using commercial available equipment [IU22^®^, S5-1 (5–1 MHz; 80 elements) probe, Philips Medical Systems©, Andover, MA, United States]. Analysis of the aortic valve and the ascending aorta was systematically performed following a dedicated protocol previously published (Goudot et al., 2019b).

### Carotid Wall Ultrafast Ultrasound Imaging

**FIGURE 1:**
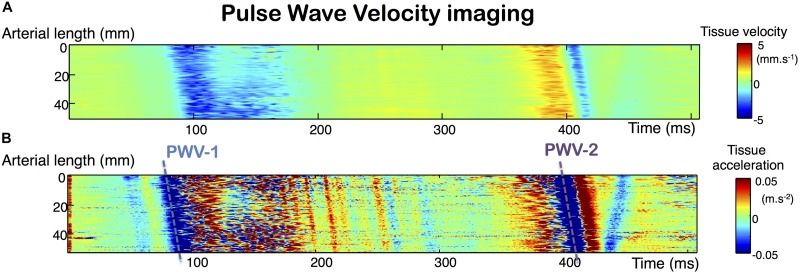
Ultrafast tissue Doppler imaging along the arterial wall over the cardiac cycle. Analysis of the tissue Doppler velocities of the arterial walls over time provides access to pulse wave velocity (PWV) measurements **(A)**. The measurement is obtained by the automated calculation of the slope of the main tissue Doppler acceleration peaks **(B)**.

**FIGURE 2:**
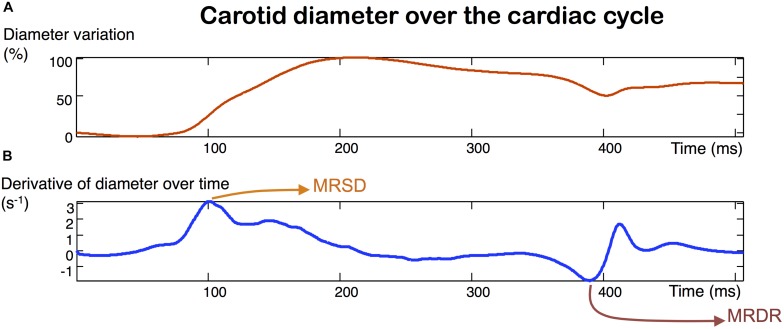
Carotid strain obtained with ultrafast ultrasound imaging **(A)**. MRSD (maximum rate of systolic distension) and MRDR (maximum rate of diastolic recoil) **(B)** are derived from the measurement of the diameter variation over time.

Bilateral evaluation of common carotid arteries by UF was performed using an Aixplorer© device (Supersonic Imagine©, Aix-en-Provence, France) and a linear probe (15–4 MHz, 256 elements, 0.2 mm pitch). The ultrafast acquisition was carried out using plane wave successively emitted at three tilted angles (–5°, 0°, and 5°), with a frame rate of 2,000 s^–1^. The acquisitions duration was 507 ms, and was triggered at the start of the QRS. The maximum depth of the image, between 10 and 40 mm was set by the operator, depending on the image obtained with conventional B-mode. Data analysis was performed using MatLab^®^ software (Version R2013b, The MathWorks©, Natick, MA, United States). For each transmitted plane wave, the backscattered ultrasonic echoes were recorded and assembled to reconstitute the 2D image using conventional delay-and-sum beamforming and spatial compounding (Montaldo et al., 2009). Tissue Doppler velocities (Figure 1A) were obtained at each point of the image at a high frame rate (Movie in Supplementary Video S1). The anterior and posterior walls of the acquisitions of adequate quality were manually segmented by an independent observer, following the signal of the carotid wall on the B-mode image obtained with UF (Supplementary Figure S1). From the initial manual delimitation of the carotid artery, the arterial diameter variation rate curve was automatically determined by calculating the difference in tissue Doppler velocities between the anterior and posterior walls over the cardiac cycle. The arterial diameter variation curve was then obtained from temporal integration of the variation rate during the cardiac cycle (Figure 2A).

#### Distensibility

Carotid distensibility was calculated according to the following formula:

$$D\,i\,s\,t=2\times \frac{S\,D-D\,D}{D\,D\times \left(S\,B\,P-D\,B\,P\right)}$$

Dist, Distensibility (mmHg^–1^); SD, Systolic diameter (mm); DD, Diastolic diameter (mm); SBP, Systolic blood pressure (mmHg); and DBP, Diastolic blood pressure (mmHg).

An oscillometric device measured brachial blood pressure. Measurements were performed on both arms and the higher value was selected. The diameter variation curve was obtained at each transversal section of the artery.

#### Pulse Wave Velocity (PWV)

The time shift of the diameter variation curve from the proximal part of the carotid to the distal part allowed us to measure the velocity of each component of the pulse wave, as suggested and measured by Couade et al. (2011):

(1) PWV 1 is the propagation velocity of the foot of the pulse wave, corresponding to the displacement of the main acceleration peak of the arterial diameter increase (Figure 1B).
(2) PWV 2 is the propagation velocity of the dicrotic notch, corresponding to the displacement of the main acceleration peak of the arterial diameter recoil (Figure 1B).

The precise measurement of each of these PWV components was based on the diameter acceleration signal (derived from the speed at which the wall moves over time). We were thus able to better identify the precise movement of the same signal along the arterial wall. For each acceleration signal, identified as corresponding to PWV 1 or 2 (Figure 1B), a linear regression of the space-time coordinates of the acceleration peaks was performed and the PWV was determined by calculating the slope of the regression. Average of right and left carotid values were done for further analyses. The ability of ultrafast ultrasound imaging to accurately evaluate the velocity of an elastic wave propagating at several meters per second within the arterial wall has been validated *in vitro* using the shear wave elastography with the same scanner used in our study (Couade et al., 2010; Maksuti et al., 2016). As well as with the shear wave elastography, PWV measurement is performed using tissue Doppler imaging on the entire length of the probe to track precisely the propagation of the pulse wave along the arterial segment and has already been used in human studies (Mirault et al., 2015; Marais et al., 2019).

#### Arterial Stiffness and Stiffening Biomarkers

Ultrafast ultrasound imaging acquisitions give access to the measurement of several complementary arterial stiffness indicators. At the carotid level, the longitudinal view of the carotid artery allowed us to measure the PWV 1, generated at diastolic arterial pressure (DBP) and PWV 2, at the time of the systolic arterial pressure peak (SBP). Since the PWV value is dependent on the level of blood pressure, each PWV value is shown divided by its corresponding blood pressure. Distensibility, maximum rate of systolic distension (MRSD) and maximum rate of diastolic recoil (MRDR), as defined by Aquaro et al. (2011) with MRI, can be measured in the same sequence, by analyzing the change in vessel diameter, obtained by tracking the wall with ultrafast tissue Doppler (Figure 2B).

### Carotid Flow Velocities and Wall Shear Stress (WSS)

To evaluate the WSS along the arterial wall, the same sequences were processed to produce vector flow images of the arterial flow. An adaptive spatiotemporal singular value decomposition (SVD) clutter filter was then used to separate tissue from blood (Baranger et al., 2018). Tissue Doppler was computed in order to track automatically the wall over the entire sequence and thus avoid WSS discrepancies due to arterial motion during the cardiac cycle. Multi-directional (–10°, 0°, 10°) color-Doppler images were assessed using sub-aperture beamforming, and used to compute flow vectors inside the lumen area. Lastly, considering blood as a Newtonian fluid, the WSS was derived using the following formula:

$$\text{τ}=\text{μ}.{||\vec{\nabla }\times \vec{\text{v}}||}_{\partial \text{Ω}}=\text{μ}.{\left|-\frac{\partial {\text{v}}_{\text{x}}}{\partial \text{z}}+\frac{\partial {\text{v}}_{\text{z}}}{\partial \text{x}}\right|}_{\partial \text{Ω}}$$

With τ(Pa): the Wall Shear Stress, μ (Pa.s): the blood viscosity, $\vec{\nabla }$ : the gradient operator, $\vec{\text{v}}\left(\text{m}.{\text{s}}^{-1}\right)$: the blood velocity, Ω describes the region of the lumen and ∂Ω its boundary.

### Statistical Analysis

Continuous data are presented as a mean ± standard deviation. Comparisons were done using a Student *t*-test. To assess the correlation between age and arterial stiffness parameters, we used the linear correlations with Pearson’s coefficients and covariance analysis (ANCOVA), according to BAV status. The categorical variables were compared using the Chi-square test. Statistical significance was considered at the 0.05 level. Analyses were performed using R^®^ software (R-Studio, version 3.4.1, Boston, MA, United States).

## Results

### Population

Ninety-two non-operated patients with BAV and forty-eight controls were consecutively assessed for their carotids in addition to their cardiac evaluation. Patients’ characteristics are presented in Table 1. BAV patients only differed from controls on gender, aortic diameters and stiffness. Men were more represented in BAV patients than in controls.

**TABLE 1:** Characteristics of BAV patients and controls.

	**BAV patients*N* = 92**	**Controls*N* = 48**	***p***
Age (years)	47.5 ± 16.6	42.6 ± 17.6	0.107
Men	62 (67)	18 (38)	<0.001
Sinus of Valsalva diameter (mm)	35.3 ± 6.6	27.8 ± 5.8	<0.001
Tubular ascending aorta diameter (mm)	36.6 ± 8.3	28.4 ± 4.6	<0.001
Mean carotid arterial diameter (mm)	6.84 ± 0.82	6.56 ± 0.82	0.066
Sinus of Valsalva distensibility (10^3^.mmHg^–1^)	1.86 ± 1.66	3.65 ± 2.19	<0.001
Tubular aorta distensibility (10^3^.mmHg^–1^)	2.70 ± 2.11	3.69 ± 2.21	0.018
DBP (mmHg)	71.5 ± 10.7	69.8 ± 10.1	0.370
SBP (mmHg)	120.2 ± 16.3	117.6 ± 15.0	0.362
PP (mmHg)	48.6 ± 12.5	47.5 ± 10.8	0.616

*Results are mean ± standard deviation, or number (percentage). BAV, bicuspid aortic valve; DBP, diastolic aortic pressure; SBP, systolic aortic pressure; PP, pulse pressure. *P*-values are from Student *t*-test and Chi-square test for sex comparison.*

### Aortic Stiffness in BAV and Controls

Diameters were larger at the sinus of Valsalva and at the tubular level of the ascending aorta in BAV patients compared with controls. It is important to note that the systolic and diastolic blood pressure values, key elements for the interpretation of the arterial stiffness indicators, did not differ between the two groups. Segmental aortic distensibility, measured by conventional echocardiography, was significantly lower in case of BAV (*p* < 0.001 at the sinus of Valsalva level and *p* = 0.018 at the tubular aortic level, Table 1).

### Carotid Stiffness Between Patients With BAV and Controls

**FIGURE 3:**
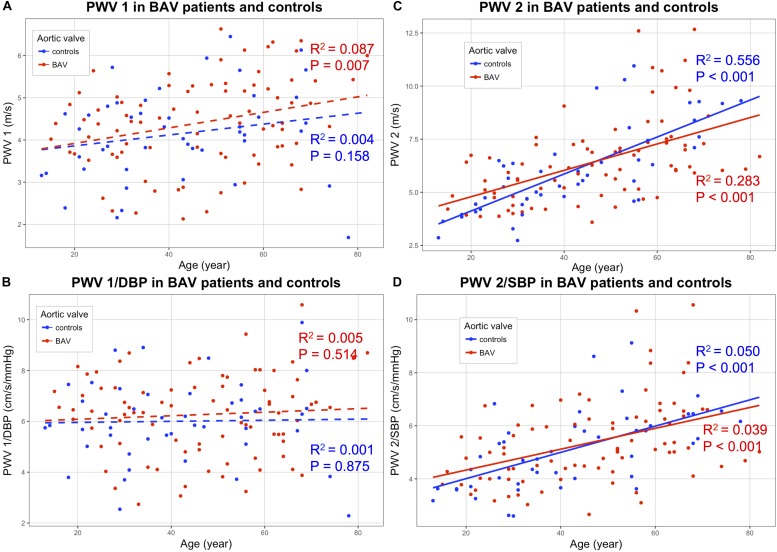
Correlations of following stiffening indicators (*Y*-axis) with age (*X*-axis): PWV 1 **(A)**; (PWV1/DBP) **(B)**; PWV2 **(C)** and PWV2/SBP **(D)**, for patients with bicuspid aortic valve (BAV) (red) and controls (blue). BAV, bicuspid aortic valve; PWV, pulse wave velocity; DBP, diastolic blood pressure; SBP, systolic blood pressure.

**FIGURE 4:**
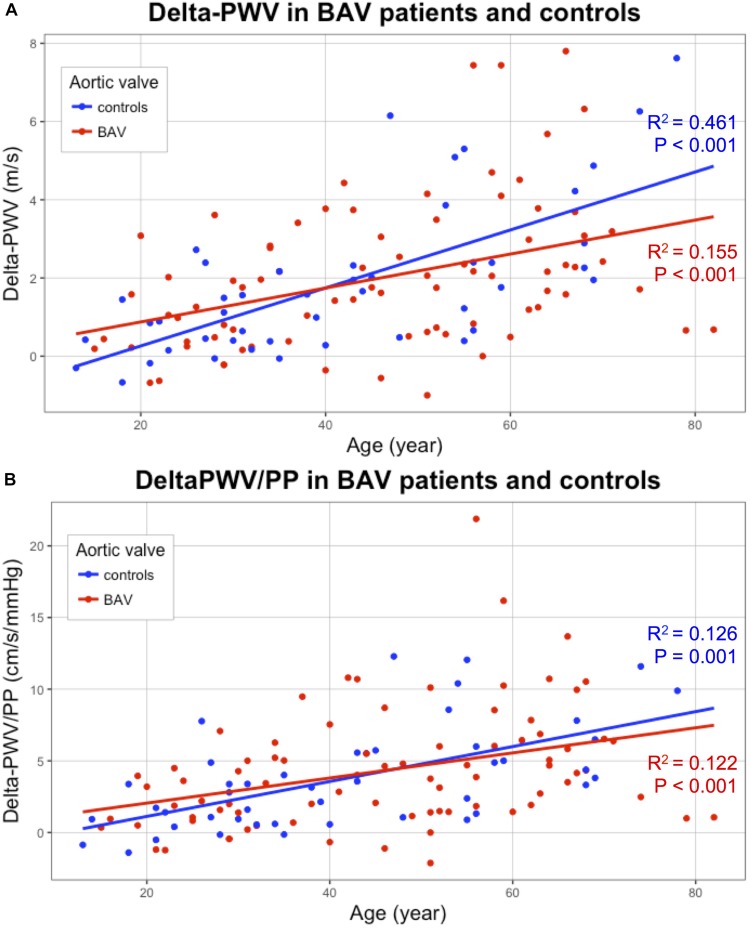
Correlation of carotid stiffening indicators (*Y*-axis) with age (*X*-axis): Delta-PWV **(A)** and Delta-PWV/PP **(B)**, for patients with bicuspid aortic valve (BAV) (red) and controls (blue). BAV, bicuspid aortic valve; PWV, pulse wave velocity; PP, pulse pressure.

**FIGURE 5:**
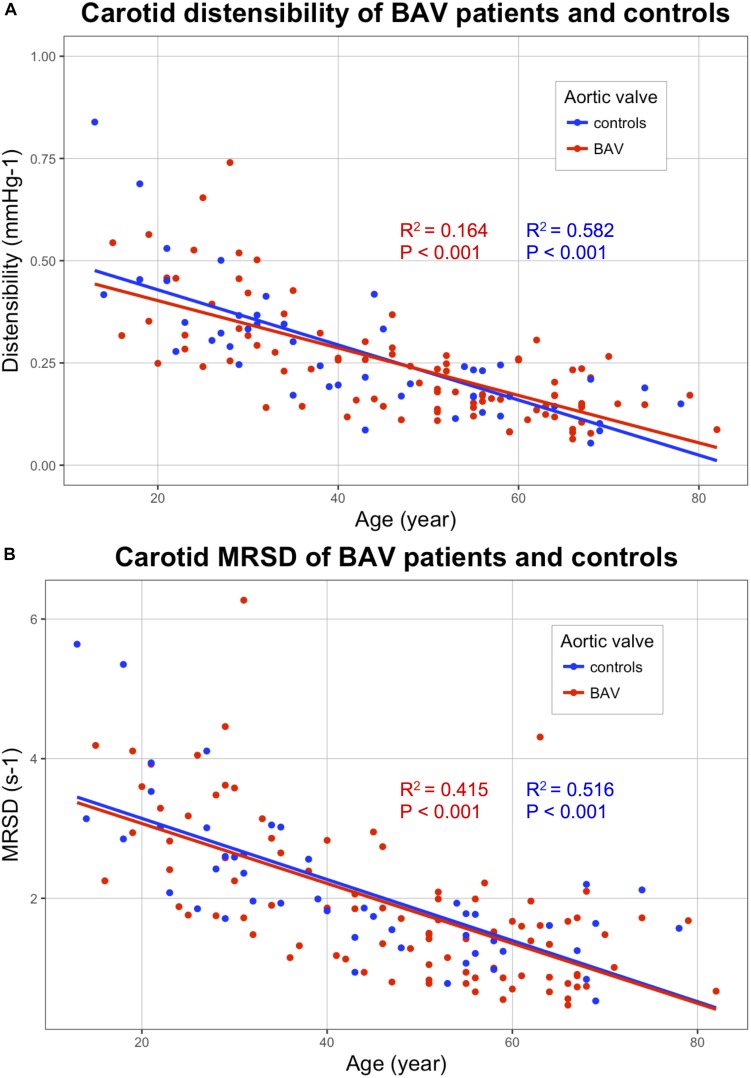
Correlations of the following stiffness indicators (*Y*-axis) with age (*X*-axis): Distensibility **(A)** and MRSD **(B)**, for patients with bicuspid aortic valve (BAV) (red) and controls (blue). BAV, bicuspid aortic valve; MRSD, maximal rate of systolic distension.

The carotid stiffness parameters, PWV raw values as well as values divided by the corresponding blood pressure, as reported by Mirault et al. (2015) are presented in Table 2. In univariate analysis, no significant difference was found between patients with BAV and controls for each carotid stiffness parameter (Table 2). Due to the influence of age on the arterial stiffness, correlations between stiffness indicators (*Y*-axis) and age (*X*-axis) are shown in Figures 3–5. A poor correlation was found for PWV1 with age (*R*^2^ = 0.087 for BAV and *R*^2^ = 0.004 for controls), but no correlation for PWV 1/DBP with age (*R*^2^ = 0.005 for BAV and *R*^2^ = 0.001 for controls) (Figure 3). Greater correlations with age were established for PWV 2 (*R*^2^ = 0.283 for BAV and *R*^2^ = 0.556 for controls), and to a lesser extend for PWV 2/SBP (*R*^2^ = 0.050 for BAV and *R*^2^ = 0.039 for controls). A significant correlation was found between arterial stiffening over the cardiac cycle, measured by Delta-PWV and Delta-PWV/PP, and age (Figure 4).

**TABLE 2:** Carotid stiffness parameters of BAV patients and controls.

	**BAV patients*N* = 92**	**Controls*N* = 48**	***p***
PWV-1 (m.s^–1^)	4.40 ± 1.06	4.14 ± 1.07	0.188
PWV-2 (m.s^–1^)	6.42 ± 1.98	6.02 ± 2.05	0.293
Delta–PWV (m.s^–1^)	2.01 ± 1.87	1.88 ± 1.92	0.723
PWV-1/DBP (cm.s^–1^.mmHg^–1^)	6.82 ± 1.47	6.38 ± 1.18	0.084
PWV-2/SBP (cm.s^–1^.mmHg^–1^)	5.29 ± 1.63	5.24 ± 1.39	0.660
Delta–PWV/PP (cm.s^–1^.mmHg^–1^)	2.87 ± 3.08	3.38 ± 2.81	0.353
Distensibility (mmHg^–1^)	26.1 ± 21.0	27.6 ± 15.6	0.503
MRSD (s^–1^)	1.89 ± 1.10	2.15 ± 1.07	0.177
MRDR (s^–1^)	1.00 ± 0.59	1.01 ± 0.52	0.942

*Results are mean ± standard deviation. BAV, bicuspid aortic valve; DBP, diastolic aortic pressure; SBP, systolic aortic pressure; PP, pulse pressure; PWV, pulse wave velocity; MRSD, maximal rate of systolic distension; MRDR, maximal rate of diastolic recoil. *P*-values are from Student *t*-test.*

Carotid diameters did not differ in BAV patients compared with controls. Concerning the indicators based on the carotid diameter variation (distensibility, MRSD, and MRDR), the correlations obtained with age were much better than those obtained by measuring the PWV. MRSD and MRDR values correlated well with age (Figure 5). No significant difference was found in any of the parameters evaluated between patients with BAV and controls (Table 2).

### Carotid Flow Parameters Between Patients With BAV and Controls

No difference in peak systolic velocity was observed between BAV patients and controls. The measurement of the WSS over the cardiac cycle allowed us to determine the maximal peak of WSS as well as the average of WSS over the whole sequence (time average WSS). There was no significant difference between BAV patients and controls for each of these parameters (Table 3, Supplementary Figure S2, and Supplementary Table S1).

**TABLE 3:** Flow parameters of BAV patients and controls.

	**BAV patients*N* = 92**	**Controls*N* = 48**	**p**
Maximal WSS (Pa)	1.80 ± 0.46	1.86 ± 0.44	0.497
Time average WSS (Pa)	0.95 ± 0.23	0.95 ± 0.20	0.870
PSV (cm.s^–1^)	116 ± 36	124 ± 39	0.266

*Results are mean ± standard deviation. BAV, bicuspid aortic valve; WSS, wall shear stress; PSV, peak systolic velocity. *P*-values are from Student *t*-test.*

## Discussion

The multiparametric analysis of carotid stiffness and carotid stiffening over the cardiac cycle did not provide any specific biomechanical change of the common carotid arteries in BAV patients, although they had a segmental aortopathy compared with controls. The study of carotid walls in case of BAV has been poorly conducted and the results presented in the literature are contradictory. A lower carotid distensibility was found using conventional ultrasound imaging by Li et al. (2016). The carotid distensibility, evaluated with B mode by the authors, remains however, a variable measurement with a low reproducibility, since it is difficult to follow precisely the carotid walls and determine exactly the minimum and maximum diameters. Kim et al. (2017) did not find any difference in carotid morphology (diameter and thickness intima-media), distensibility and peak tissue velocity using tissue Doppler on the carotid wall. This technology may be more accurate than the classic B-mode to assess distensibility. The tissue Doppler allows indeed a more precise measurement of small displacements of the arterial wall because it is based on a very precise estimate of the displacement velocity. It allows to quantify sub-wavelength displacements (much less than the mm) which is not possible with the speckle tracking (Bonnefous and Pesqué, 1986).

Our results converge toward the absence of the common carotid artery involvement in BAV-associated vascular remodeling, which appears to affect only the ascending aorta, and particularly the sinus of Valsalva (Goudot et al., 2019b). From an embryological point of view, the smooth muscle cells forming the aortic valve and the sinus of Valsalva come from the second cardiac field, while the smooth muscle cells forming the tubular aorta and the common carotid arteries come from the neural crest (Yassine et al., 2017). This divergent embryological lineage could explain the results obtained on all the vascular walls studied: on the one hand, a more important stiffness of the sinus of Valsalva appearing at a younger age, with a stiffness acquired later, dependent on dilatation of the tubular aorta, and, on the other hand, no damage to the common carotid arteries nor to the abdominal aorta (Goudot et al., 2019b). New technical settings are mandatory to be able to assess the ascending aorta by transthoracic ultrafast ultrasound imaging. The evaluation of similar morphological biomarkers with ultrasound would provide a simple and easily accessible tool, which could be rapidly integrated into the follow-up of patients with BAV, usually performed only by echocardiography. The aortic stiffness could thus be an additional measure to the usual measures of the aortic diameters at the different segments of the ascending aorta (Erbel et al., 2014).

Lastly, we provide a technological innovation regarding the combined evaluation of WSS and carotid stiffness. Hemodynamic assessment of blood flow is an important factor in the arterial wall remodeling (Yassine et al., 2017). The lack of WSS change in case of BAV could thus explain the absence of stiffness modification of the carotid wall. WSS modifications in the ascending aorta related to the specific morphology of the BAV appear to be indeed responsible for a significant proportion of degenerative lesions observed in the aortic wall (Guzzardi et al., 2015). Developing ultrasound imaging combining stiffness and WSS measurements would therefore be particularly relevant to assess aortopathy associated with BAV.

### Limitations

All the stiffness data were only processed after examining the patient. The distensibility is indeed not currently available on the commercialized UF ultrasound scanner. The automated method for collecting stiffness parameters in real time could be however, quickly implemented. The study of first-degree relatives as control patients is also a limitation. BAV first-degree relatives cannot indeed be considered completely free from minor aortic involvement. In our series, even though we have shown no difference in carotid stiffness, there were, however, significant differences in diameter and aortic distensibility between BAV patients and controls.

## Conclusion

Ultrafast ultrasound imaging allows an automated simultaneous evaluation of carotid stiffness and flow parameters over the cardiac cycle. No difference was found for stiffness and stiffening parameters between BAV patients and controls at the common carotid level. An aortic evaluation, using a dedicated phased array probe, would provide the parameters of aortic distensibility, MRSD, and PWV. This study is progressing, with a specific sequence for a phased array probe currently being developed.

## Data Availability Statement

The datasets generated for this study are available on request to the corresponding author.

## Ethics Statement

The studies involving human participants were reviewed and approved by CPP Île-de-France VI (2017-A01508-45). Written informed consent to participate in this study was provided by the participants’ legal guardian/next of kin.

## Author Contributions

GG, CC, and LK carried out the dedicated consultations and collected the data. OP, JP, MG, and MP analyzed the data. GG and TM performed the statistical analysis and wrote the manuscript. MP, XJ, and EM proofread the manuscript. EM organized this study, supervised the cardiac ultrasounds, performed final approval of the version to be published, and agreed to be accountable for all aspects of the work in ensuring that questions related to the accuracy and integrity of any part of the work are appropriately investigated and resolved.

## Conflict of Interest

The authors declare that the research was conducted in the absence of any commercial or financial relationships that could be construed as a potential conflict of interest.

## Supplementary Material

FIGURE S1 Longitudinal section of a common carotid artery with ultrafast ultrasound B-mode imaging. The segmentations of the anterior (blue line) and posterior (red line) walls are indicated in the picture.

FIGURE S2 Correlations of the wall shear stress (WSS) parameters (*Y*-axis) with age (*X*-axis): Maximal WSS **(A)** and time-average WSS **(B)**, for BAV patients (red) and controls (blue). BAV: bicuspid aortic valve. Pearson’s coefficients square (*R*^2^) and *P*-values for each correlation line are presented.

TABLE S1 Linear correlations of maximal wall shear stress (WSS) and time average WSS, with age.

VIDEO S1 Ultrafast ultrasound imaging of a common carotid artery. Representation of the tissue Doppler data (color scale). The propagation of the pulse wave components (PWV1 and PWV2) is notified on the ECG recording.
