# Supplementary figures and images for: Carotid Stiffness Assessment With Ultrafast Ultrasound Imaging in Case of Bicuspid Aortic Valve
Source: Front Physiol. 2019 Oct 23;10:1330. doi: 10.3389/fphys.2019.01330 (PMC6819321; doi:10.3389/fphys.2019.01330)

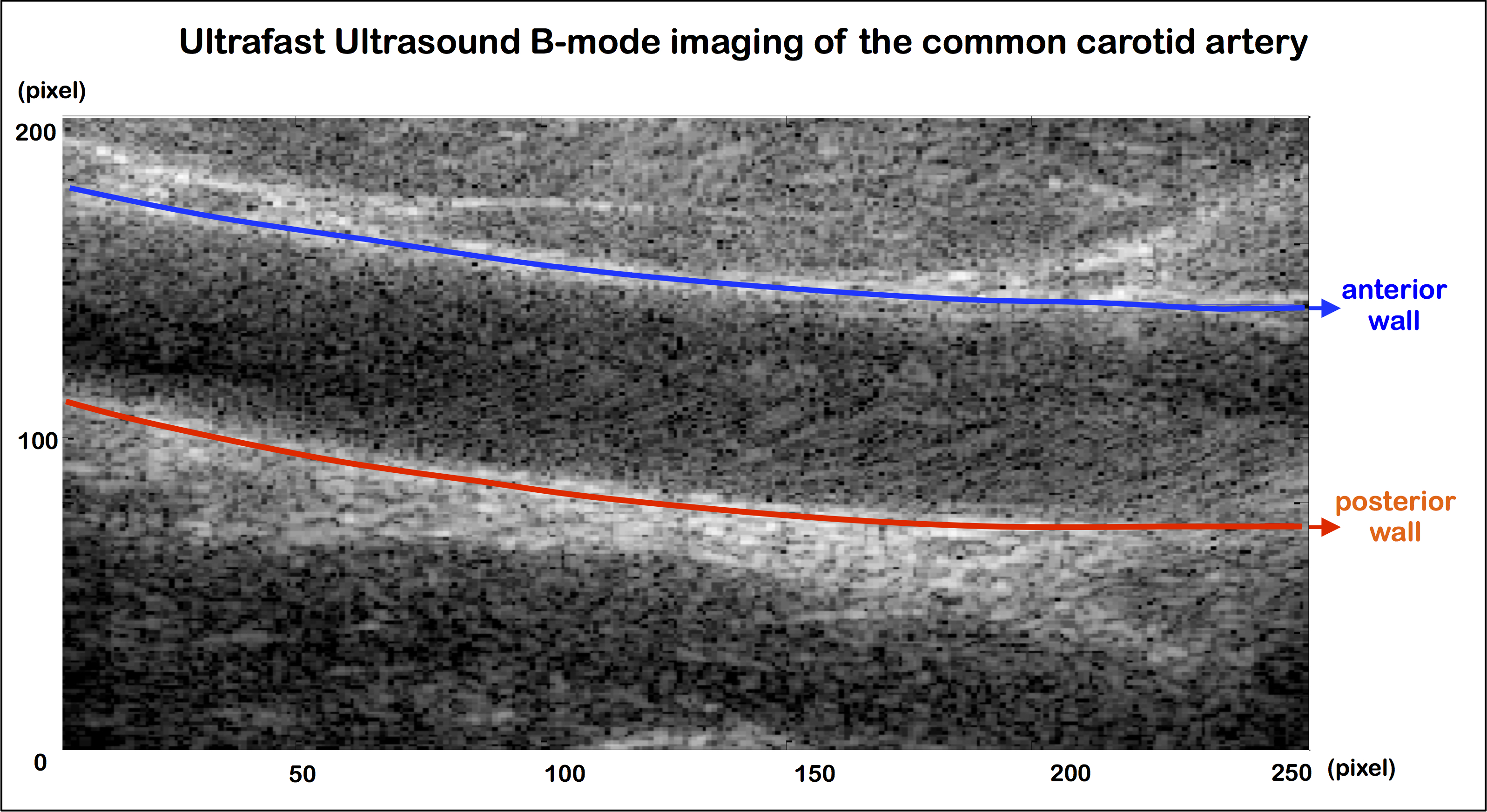

Supplement: FIGURE S1 — Longitudinal section of a common carotid artery with ultrafast ultrasound B-mode imaging. The segmentations of the anterior (blue line) and posterior (red line) walls are indicated in the picture. [file Image_1.JPEG]

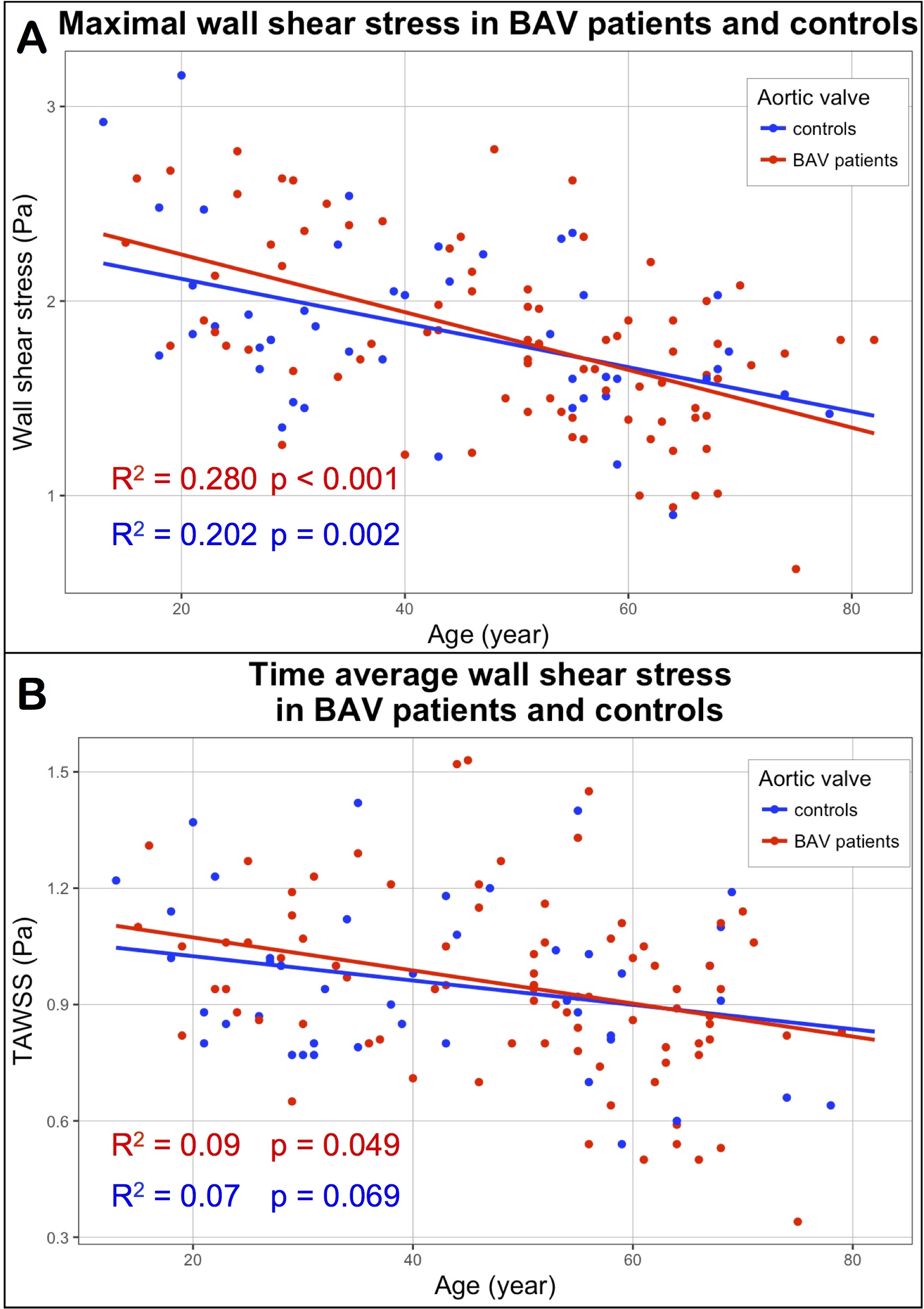

Supplement: FIGURE S2 — Correlations of the wall shear stress (WSS) parameters (Y-axis) with age (X-axis): Maximal WSS (A) and time-average WSS (B), for BAV patients (red) and controls (blue). BAV: bicuspid aortic valve. Pearson’s coefficients square (R2) and P-values for each correlation line are presented. [file Image_2.JPEG]
