# Supplementary material for: Carotid Stiffness Assessment With Ultrafast Ultrasound Imaging in Case of Bicuspid Aortic Valve
Source: Front Physiol. 2019 Oct 23;10:1330. doi: 10.3389/fphys.2019.01330 (PMC6819321; doi:10.3389/fphys.2019.01330)
Supplement: TABLE S1 — Linear correlations of maximal wall shear stress (WSS) and time average WSS, with age. [file Table_1.docx]

**Supplementary Table S1.** Linear correlations of maximal wall shear stress (WSS) and time average WSS, with age. Pearson’s coefficients square (R^2^), P-values, and the slope of correlation lines (a) are presented. P-ancova value is obtained by covariance analysis between the BAV group and the controls.

| Y-axis | X-axis | BAV  n = 92 | | | Controls  n = 48 | | | P-ancova |
| --- | --- | --- | --- | --- | --- | --- | --- | --- |
|  |  | R^2^ | a | p | R^2^ | a | p |  |
| Maximal WSS | Age | 0.280 | -0.015 | < 0.001 | 0.202 | -0.011 | 0.002 | 0.609 |
| Time average WSS | Age | 0.093 | -0.004 | 0.049 | 0.073 | -0.003 | 0.069 | 0.612 |
